# Hypoxia-driven splicing into noncoding isoforms regulates the DNA damage response

**DOI:** 10.1038/npjgenmed.2016.20

**Published:** 2016-07-20

**Authors:** Danish Memon, Keren Dawson, Christopher SF Smowton, Wei Xing, Caroline Dive, Crispin J Miller

**Affiliations:** 1 RNA Biology Group, CRUK Manchester Institute, The University of Manchester, Manchester, UK; 2 Scientific Computing Team, CRUK Manchester Institute, The University of Manchester, Manchester, UK; 3 Clinical and Experimental Pharmacology Group, CRUK Manchester Institute, The University of Manchester, Manchester, UK

## Abstract

Tumour hypoxia is associated with poor patient outcome and resistance to therapy. It is accompanied by widespread changes in gene expression mediated largely through the transcription factors HIF1/2/3α. Hypoxia impacts on multiple pathways throughout the cell and has widespread effects on phenotype. Here we use sample-specific annotation approaches to determine the changes in transcript architecture that arise as result of alternative splicing in hypoxic cells. Using *in vivo* data generated from a time course in reduced oxygenation we identified genome-wide switching between coding and noncoding isoforms, including a significant number of components of the DNA damage response pathway. Notably, HDAC6, a master regulator of the cytotoxic response, and TP53BP1, which sits at the nexus of the double-strand break repair pathway, both underwent a marked transition towards an intron-retention pattern with a concomitant decline in protein levels. These transitions from coding to noncoding isoforms were recapitulated in a large and independent cohort of 499 colorectal samples taken from The Cancer Genome Atlas (TCGA). The set of altered genes was enriched for multiple components of the Fanconi Anaemia, nucleotide excision and double-strand break repair pathways, and together correlating with tumour status at last contact. Altogether, these data demonstrate a new role for hypoxia-driven alternative splicing in regulating DNA damage response, and highlight the importance of considering alternative splicing as a critical factor in our understanding of human disease.

## Introduction

Hypoxia occurs within the majority of solid tumours and is associated with poor patient outcome and chemo- and radioresistance.^1,2^ Hypoxia arises both because disorganisation within tumour microvasculature lengthens intracapillary distances beyond the diffusion range of oxygen and because transient disruptions to blood flow provoke periods of acute oxygen starvation. Hypoxia has multiple impacts on tumour biology including selection of altered cell signalling, angiogenesis, vasculogenesis, changes in central metabolism, suppression of immune reactivity, enhanced receptor tyrosine kinase signalling and down regulation of DNA repair pathways, promotion of prosurvival phenotypes and increased proclivity for invasion and metastasis;^3,4^ extensively reviewed in references 5,6. Many of these hypoxia responses are characterised by widespread alterations in transcription profiles driven largely (but not exclusively) by stabilisation of the transcription factor subunit hypoxia inducible factor 1α (HIF1α).^7,8^ Hypoxia mediated transcriptional regulation is also controlled by other factors including HIF2α^9^ and HIF3α.^10^ In addition, signalling through both the growth factor receptor pathways (phosphatidylinositol 3-kinase; PI3K, ERK) and energy depletion pathways (5-AMP-activated protein kinase; AMPK) converge on the tuberous sclerosis complex (TSC1/2) leading to complex patterns of spatial and temporal regulation in response to stress. These signals feed into mechanistic target of rapamycin (mTOR), which, in the context of hypoxia, leads to the rapid suppression of protein synthesis,^11^ presumably in order to conserve energy.^12^ Levels of hypoxia vary between and within tumours, correlate with patient outcomes, and can lead to differences in response to therapy.^13^ A better understanding of heterogeneity in hypoxia-driven changes in gene expression will therefore inform strategies for precision medicine,^14^ raising the need for reliable biomarkers of tumour hypoxia. To this end, a number of groups have developed multiplex gene expression signatures with the intention of better reflecting the multiplicity of pathways involved in the hypoxic response, e.g., references 13,15,16.

In recent years, advances in expression profiling have revealed substantial levels of alternative splicing within the human genome, such that the majority of protein-coding genes are now known to express multiple isoforms (median isoform count per protein coding gene in ENSEMBL 74: 5), of which 44% are annotated as noncoding (63,816: ‘noncoding’; 81,715: ‘protein-coding’). Despite their prevalence, the majority of these transcripts have yet to be characterised, raising the question of how much of this ‘dark matter’ is functional, and how much is simply a consequence of aberrant splicing and a passive by-product of gene expression.

Given the widespread alterations in transcript expression that arise in response to changes in oxygen levels, we speculated that similar systematic alterations in splicing might add further levels of transcriptional control. We therefore exploited the increased precision offered by RNA sequencing to investigate how hypoxia affects alternative splicing, since earlier studies using 3′ IVT arrays were not able to characterise the transcriptome at this level of precision. We used *de novo* sample-specific annotation strategies to investigate changes in exon structure alongside multiple events including intron retention and alterations to 5′ and 3′ gene boundaries. We applied these novel annotation approaches both to a time course of colorectal cancer cells in reduced oxygen and to reanalyse a large cohort of colorectal samples from TCGA.

## Results

### Systematic resplicing of the transcriptome in response to hypoxia

RNA deep sequencing over a time course of enforced hypoxia was used to identify changes in coding and noncoding RNA levels in HCT116 cells. Cells were collected at 0, 1, 2, 24 h in reduced oxygen (1% O_2_) and poly(A) RNA sequenced using 100mer paired-end strand-specific Illumina sequencing. FACS analysis revealed no significant effect on cell proliferation or apoptosis at 24 h in response to hypoxia (Supplementary Figure 1). Data were aligned and exon structure determined using a multistage pipeline including DEXSeq,^17^ Mapsplice^18^ and Cufflinks^19,20^ to identify significant events such as exon skipping and intron retention (Supplementary Figure 2; Supplementary Methods). This generated an augmented catalogue of transcripts in which novel isoforms identified by Cufflinks were amalgamated with existing annotations from ENSEMBL, before splicing changes were identified through Multivariate Analysis of Transcript Splicing (MATS).^21^

Of the 53,936 transcripts identified, 52,733 mapped to 15,334 genes in ENSEMBL (v74)^22^ while 1,203 (1,155 genes) were unannotated (Supplementary Table 2). In keeping with previous reports,^15^ 12% of protein-coding genes exhibited gene-level changes within the time course (*N*=2,387), and included multiple genes associated with hypoxia, glycolysis and MAPK signalling (Supplementary Table 3; Supplementary Figure 2). At the isoform level, 9,222 (60%) of genes were present in at least 2 isoforms, of which 4,982 (54%) were predicted to code for isoforms with at least one novel fragment, and therefore sharing only a subset of known splice sites (Supplementary Table 2). Multiple changes to within the coding sequence were identified using Cuffdiff. These included multiple kinases (CLK4, MARK4, ACVR2B, MAP3K3, MAP3K8, STK32C and SGK494) and 2 phosphatases (PPP5C, PPP3CB; see Supplementary Table 4; Supplementary Figure 4 which also lists loci with alternate 5′ ends).

**Figure 1:**
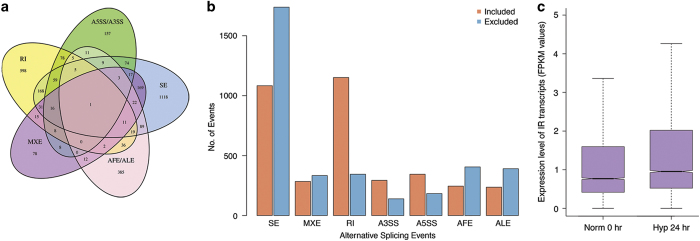
Isoform switching in hypoxia increases the abundance of unproductive transcripts. (**a**) Multiple splicing events affect the majority of alternatively spliced genes. A3SS, alternative 3′ start site; A5SS, alternative 5′ start site; AFE, alternative first exon; ALE, alternative last exon; MXE, mutually exclusive exons; RI, retained intron; SE, spliced exon. (**b**) Inclusion or exclusion of exons in response to hypoxia, detected by MATS. Categories as **a**. (**c**) Overall expression levels of genes that switch between ‘protein-coding’ and ‘retained-intron’ major isoform between normoxia and hypoxia.

Splicing events were frequent and diverse. 29% (*N*=869/2,949) of spliced genes were predicted by MATS to exhibit multiple concurrent modifications (Figure 1a), including a substantial increase in the number of retained introns, increased exon skipping, increased usage of alternate 5′ and 3′ splice sites (Figure 1b; Supplementary Table 4; Supplementary Figure 5), and a global shift towards expression of retained intron transcripts (Figure 1c). Genes involved in exon skipping and inclusion exhibited high enrichment for Cell cycle (GO:0007049) and RNA splicing (GO:0008380). The RNA splicing pathway was also enriched for genes that exhibited increased intron retention in hypoxia, suggestive of a feedback loop in which components of the alternative splicing machinery are themselves regulated in part by changes to splicing patterns. Importantly, genes associated with Response to DNA Damage Stimulus and DNA repair pathway also exhibited increased intron retention (Benjamini and Hochberg corrected P value<0.05).

### Confirmation that retained intron expression regulates expression of HDAC6 and TP53BP1 protein levels

In addition to changes between protein coding isoforms (Supplementary Figure 6), 29% of genes switched from a protein-coding to noncoding major isoform, with the majority of events (199/343) leading to the expression of a noncoding isoform as the primary transcript under hypoxia (Supplementary Table 5). Of these, most changes were driven by intron retention (88/132) and were consistent with an overall increase in the expression of retained-intron transcripts after 24 h in hypoxia (P value<0.001; Figure 1b,c).

**Figure 2:**
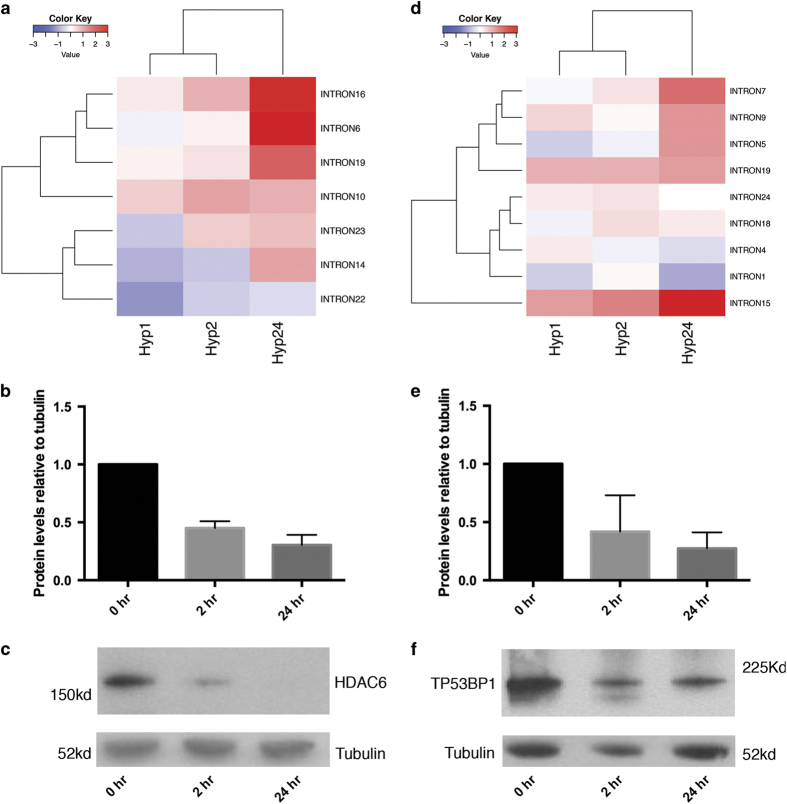
Changes in HDAC6 and TP53BP1 transcript and protein levels in response to hypoxia. (**a**) Expression across introns in HDAC6 at 0, 2, 24 h following a shift to reduced oxygen (1%). Colour represents log_2_ fold-change. (**b**) HDAC6 protein levels as determined by western blot (**c**). (**d**–**f**) TP53BP1 exhibits similar patterns of expression.

We hypothesised that this might provide a previously unreported mechanism by which cells could modulate protein levels in response to hypoxia, and therefore sought to confirm that a switch towards a noncoding isoform was indeed associated with altered protein levels. Two notable genes with substantial changes in intron retention were HDAC6 and TP53BP1 (Figure 2a,d). HDAC6 is a class IIb histone deacetylase with an unusually diverse set of substrates that include multiple cytosolic proteins such as HSP90. HDAC6 inhibition has been associated with the processing of protein aggregates, the misfolded protein stress response pathway, and more generally as a master regulator of cytotoxic stress,^23,24^ including its involvement in the ubiquitination and deacetylation of the mismatch repair (MMR) protein MutS protein homologue 2 (MSH2).^25^ Levels of intron expression at the locus increased in hypoxic conditions and were accompanied by a significant decline in protein levels (Figure 2b,c). TP53BP1 also exhibited an increase in intron retention and an associated decline in protein levels (Figure 2e,f). It sits at the nexus of the double-strand break repair pathway where it performs a number of roles, including binding to P53, leading to enhanced transactivation and increased levels of P21,^26^ interacting with chromatin to promote DNA repair,^27^ and recognising H4K20me2 and H2AK15ub histone marks arising from double-strand break signalling.^28^ TP53BP1 has also been shown to cooperate with RIF1 and MAD2L2 (Rev7)^23^ to modulate nonhomologous end joining and genetic stability. These data are therefore particularly interesting in the context of a ‘mutator phenotype’ in which a shift to increased genetic instability is postulated to promote clonal diversity within a tumour.^24^

### Colorectal tumours express high proportion of unproductive transcripts

**Figure 3:**
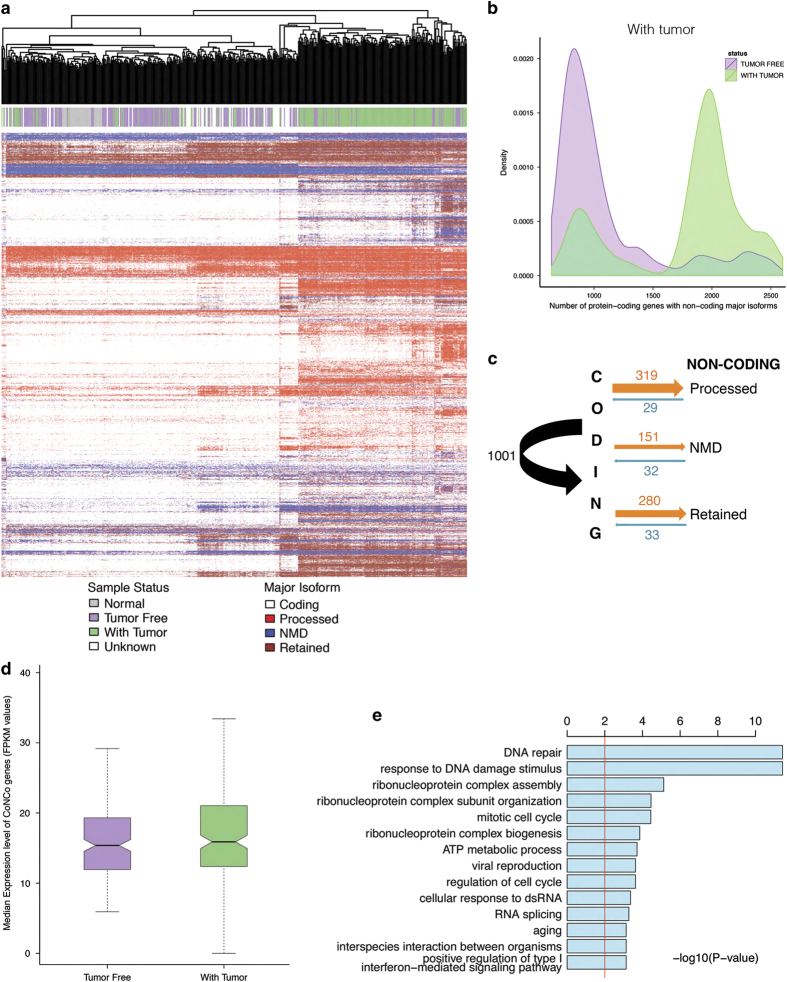
Switch from coding to noncoding transcripts is a signature of colorectal tumours. (**a**) Change in major isoform class between ‘tumour-free’ and ‘with tumour’ samples in TCGA colorectal carcinomas; Orange: enriched in ‘with tumour’ samples. (**b**) Unsupervised clustering of colorectal tumour samples based on major isoform type: NMD, ‘nonsense-mediated decay’; Processed, ‘processed transcript’; RI, ‘retained Intron’. (**c**) Proportion of noncoding major isoforms detected across colorectal tumour samples stratified by patient status at last contact: ‘tumour free’ (purple) and ‘with tumour’ (green). Data from TCGA. (**d**) Average expression level of genes changing from coding to noncoding major isoform (CoNCo) stratified by patient status on last contact. (**e**) Gene ontology terms found enriched among CoNCo genes.

Having identified a new role for alternative splicing in regulating protein levels in response to hypoxia, we asked whether similar changes were observed in human tumours. We analysed RNA-seq data derived from 458 colorectal tumour samples and 41 normal tissues obtained from The Cancer Genome Atlas (TCGA).^29^ The samples included AJCC pathologic staging (Stage I–Stage IVB), metastatic staging (M0, M1 and MX) and therefore included both patients with early-localised disease as well as metastasis. No information was available about previous treatment with radiotherapy or chemotherapy for the majority of patients. As the TCGA data were processed on two different platforms (an Illumina HiSeq 2000 and an Illumina GAII), we tested whether there were significant differences in mapping rates to introns and exons between the two platforms. No inconsistencies were identified between the two platforms, and major isoforms for each gene were assigned independently for each sample, further mitigating against batch effects. In total, 4,303 genes were found to switch between coding and noncoding major isoforms in at least 5% of samples (Figure 3a; Supplementary Table 6). Unsupervised clustering of these data by major isoform coding/noncoding status revealed three major clusters (Figure 3a). The proportion of noncoding major-isoforms correlated well with available relapse data, with tumours from patients who were subsequently defined as ‘tumour free’ at last contact expressing fewer noncoding major-isoforms than those defined as ‘with tumour’ (tumour-free: 916; with tumour: 1945; *P *value <0.01; Figure 3b). As expected, many of these also switched between coding and noncoding isoforms in the HCT116 cell line data (*P* value <0.01; hypergeometric test), suggesting that a significant proportion of these changes are driven by changes in oxygenation status within the tumours. Interestingly, 750 genes changed in major-isoform status from coding to noncoding between these two classes (Figure 3c; Supplementary Table 6); however, switching to a noncoding isoform was not significantly associated with a global change in overall transcription levels associated with these loci (Figure 3d). These data highlight the importance of considering the consequences of splicing when seeking expression signatures using transcription data.

Finally, we asked whether these splicing changes in tumour samples were associated with specific pathways, and found significant enrichment for DNA damage and repair pathways, as well as alternative splicing (‘Response to DNA Damage Stimulus’, ‘DNA Repair’ and ‘RNA Splicing’ gene ontology (GO) categories; Benjamini and Hochberg corrected *P* value <0.01; Figure 3e). A significant proportion of these loci were also known downstream targets of TP53 (Ingenuity; IPA; *P *value of overlap: <10^−6^).

## Discussion

Although hypoxia-dependent splicing changes have been reported for individual loci,^30–32^ there has been no evidence on how widely applicable the phenomenon may be.^33^ Here we identified a comprehensive remodelling of transcript structures in response to hypoxia, encompassing significant changes in the levels of 12% of all protein-coding transcripts, and a switch to a different major isoform at 7% of all protein-coding loci. We observed similar changes in colorectal carcinomas and a widespread switch to noncoding isoforms that correlated strongly with tumour status at last contact. Many of these changes involved expression of novel transcripts not present in the ENSEMBL reference annotation database.

While splicing to remodel the proteome is well understood to be a critical process through which cells achieve increased protein diversity,^34^ we have identified a novel role for splicing in which switching between noncoding isoforms modulates overall protein output from a locus. Hypoxia-dependent changes were biased towards loss of the protein-coding isoform under hypoxia and consistent with the rapid decline in protein synthesis that accompanies a shift to hypoxic conditions (Figure 3). Importantly, loci that changed in this way were significantly enriched for specific pathways, including those that respond to DNA damage. The effects observed are, therefore, not simply the result of overall loss of fidelity in the splicing machinery, but rather a consequence of its coordinated reprogramming, and confirming the importance of identifying switching events in isoform expression.^35^ Altogether, these data indicate the presence of a tightly regulated pathway, and are therefore important in the context of recent reports that somatic single-nucleotide variants can result in aberrant splicing patterns, including intron retention, that deactivate tumour suppressor genes.^36^ Our data indicate that these splicing patterns may occur naturally as part of normal regulatory processes, and that single-nucleotide variants provide a mechanism by which cancer cells can hijack these pathways to subvert normal mechanisms of control. This transformation of the coding competence of the transcriptome occurs in parallel with changes to other regulatory pathways including epigenetic modifiers and multiple kinases, thus providing further opportunities to rewire cancer-signalling pathways.^37^

Both HDAC6 and TP53BP1 exhibited a significant decline in protein level concomitant with increased intron retention. These data, together with coding–noncoding splicing changes to multiple key components of the Fanconi Anemia pathway (MU31, FANCB, FANCG, FANCM, ATRIP, POLG and RPA1), the nucleotide excision repair pathway (AQR, PLD1, ERCC2, LIG1, RPA1, CCNH, RNF11, ACTL6A, RFC1, REV1, ACRT8 and GTF2H2) and the double-strand break repair pathway (RAD9, WRN, SMARCA5, UIMC1, RFC1, MUS81, POLD1, ATRIP, CHEK1, CLSPN, BABM1, TP53BP1, RPA1, REV1, CDK2 and RAD52), together reveal a hitherto unanticipated role for alternative splicing in modulating the DNA damage response through action both at critical regulators (e.g., HDAC6, TP53BP1; Figure 2 and the G2-M checkpoint kinase CHEK1) and by modulating the level of proteins throughout the pathway.

Hypoxia-dependent increases in genetic instability have previously been suggested as a driver of a ‘mutator phenotype’,^38^ in which elevated mutation rates increase clonal diversity, leading to a greater likelihood of the expression of a clone with genetic changes that confer a proliferative advantage.^24^ Taken together, our data suggest that a shift to noncoding expression in response to hypoxia may provide a novel, important, and unanticipated mechanisms by which these processes are mediated.

Our reanalysis of TCGA data also revealed a striking signature of outcome in which a switch to a noncoding major isoform at multiple protein coding loci is associated with patients annotated as ‘with tumour at last contact’. Importantly, these changes are not detected using gene-level expression summaries, as overall RNA levels from these loci do not change substantially. Our findings have clear implications for the analysis of expression data and the development of RNA-based signatures and biomarkers in both diagnostics and personalised medicine.

## Materials and methods

### Cell culture

HCT116 were cultured in RPMI-1640 media (Life Technologies, Grand Island, NY, USA) supplemented with 10% FBS (fetal bovine serum; Biosera, Sussex, UK, FB-1000/500). All cells were maintained in a humidified atmosphere at 37 °C and 5% CO_2_. For hypoxia treatment, the HCT116 cell line was cultured in 1% O_2_ in an Invivo_2_ hypoxia workstation 4000 (Biotrace, Fred Baker, Runcorn, UK) for the given time course 24 h after plating. Cells were obtained from ATCC tested and authenticated using multiplex PCR (Powerplex 21, Promega, Madison, WI, USA; 15.03.10).

### Cell cycle analysis by flow cytometry

Cells were fixed with 70% ethanol. Briefly, cells were collected and washed in PBS–2%BSA twice and resuspended to a concentration of 3–6 mill/ml. 500 ml were taken and 5 ml cold 70% ethanol was added dropwise to the cells whilst gently vortexing to prevent cell clumping. Cells were fixed at 4 °C for 1 h and stored at −20 °C until ready for FACS analysis. The cells were stained with FxCycle PI/RNase Staining Solution solution (Life Technologies F10797) according to the manufacturer’s protocol and the cells were analysed using the BD FACS Canto 11 system with the BD FACS Diva software version 8 (BD Biosciences, San Jose, CA, USA). Data analysis was performed using the Modfit4 software (Verity Software House, Topsham, ME, USA).

### Protein extraction and western blotting

Protein was extracted by washing cells in ice-cold phosphate-buffered saline (PBS) and scraping cells in ice-cold cell lysis buffer (9803s New England Biolabs, Ipswich, MA, USA) supplemented with PMSF (Sigma, St Louis, MO, USA, 93482) and protease inhibitors (Roche Diagnostics Corp., Indianapolis, IN, USA, complete EDTA free 11,873,580,001). The sample was centrifuged at 4 °C 13,000 r.p.m. for 10 min and supernatent was preserved. Total protein per sample of 50 μg was resolved by SDS-polyacrylamide gel electrophoresis 10% NuPage gels (Invitrogen, Carlsbad, CA, USA) and transferred electrophoretically to Immobilon-P (Millipore, Temecula, CA, USA). The membrane was blocked in 5%milk PBS-T for 30 min and blotted overnight with HDAC6 antibody (1/1,000 NEB 7558S), TP53BP1 (1/1,000 AT4311a Generon mouse monoclonal antibody), or tubulin antibody (1/5,000 Sigma T6199). Detection was performed using a peroxidase-conjugated anti-rabbit or anti-mouse IgG (Amersham Biosciences Pharmacia, Piscataway, NJ, USA) and chemiluminescence visualisation (ECL+, Amersham Biosciences) was used according to the manufacturer’s instructions. Quantification of western blot signals was performed using the Chemi Genius Bioimaging system (Syngene, Frderick, MD, USA) and the Chemi genius gel documentation and analysis system.

### RNA extraction and construction of sequencing libraries

RNA was extracted using the Qiagen Qiashredder kit (79654) and the Qiagen RNeasy Mini Kit (74104) as per the manufacturer’s instructions (Qiagen Inc., Valencia, CA, USA). The RNA was DNase treated following the protocol in the RNeasy Mini Kit with Qiagen RNase-free DNase I (79254). Indexed PolyA libraries were prepared using 1 μg of total RNA and 13 cycles of amplification in the NEB Next Ultra Directional RNA Library Prep Kit for Illumina (New England Biolabs, Cat No: E7420S). RNA Integrity Values scores were >9 for all samples. Libraries were quantified by qPCR using a Kapa Library Quantification Kit for Illumina sequencing platforms (Kapa Biosystems, Wilmington, MA, USA, Cat No: KK4835). Pooled libraries were clustered at 15 pmol/l on the cBot and 2×100 bp sequencing was carried out using the High Throughput mode of a HiSeq 2500 using TruSeq SBS Kit v3 chemistry (Illumina)

### Data analysis

All statistical analysis including *t*-tests and Wilcoxon’s tests, were performed in R. Time-course data: 100mer paired reads were aligned to the human genome (hg19) using Mapsplice (v2.1.4; default parameters),^18^ which has previously been shown to perform well for *de novo* splice junction identification.^39^ An average of 43.8 M (27.5–58.2 M) read-pairs per sample mapped to the genome in the correct orientation and appropriately spaced, corresponding to ~90% of the total reads sequenced. Alternative splicing analysis can be affected by contamination from premature and pre-mRNA transcripts in the poly(A) selected pool. This is manifested by increased numbers of reads mapping to introns. We calculated the read distribution in different regions including exons, introns, untranslated regions, promoters and intergenic regions derived from the genomic annotations in Ensembl (hg19), and found the majority of reads fell within exons and untranslated regions, whereas only a very small proportion of reads mapped to introns. Further, these data were consistent across the samples and there were no significant differences in these overall QC values between replicate groups (Supplementary Table 1). Transcript models were derived for each sample independently using Cufflinks (v2.2.0; with default parameters, except to specify strand specificity; https://github.com/cole-trapnell-lab/cufflinks). Resultant models were then merged using Cuffmerge to provide a global model and to classify transcripts as novel, or known, when they mapped to ENSEMBL (v74; http://www.ensembl.org). In order to minimise false positives, a deliberately stringent filtration was used in order to call novel transcripts: Gene models were first filtered to keep transcripts only when an exon junction was supported by at least 2 reads in at least two samples. This filtration step removed a large proportion of potentially unreliable exon junctions, such that following this step 68.2% of exon junctions completely matched Ensembl annotations, whereas 30% differed in either the start or end site but not both and only 1.8% of which were completely novel (Supplementary Table 1). The transcripts were also filtered for overall normalised expression levels, with only transcripts with fragments per kilobase mapped (FPKM) >0.5 in at least three samples being retained for analysis. These transcripts were subsequently classified according to transcript type and provided in the Supplementary Table 2. The 53,936 transcripts (15,334 genes) were used to obtain gene-level counts using the RsubRead package^40^ in R and supplied to edgeR^41^ to call differential expression (absolute fold-change >2 relative to 0 h; false discovery rate (FDR)<1%) at each time point. Annotation was supplied by the Bioconductor package annmap.^42^

### Gene ontology enrichment analysis

Functional enrichment analysis was performed using the Goseq package^43^ in R to identify statistically enriched GO terms^44^ (Hypergeometric test: Benjamini and Hochberg corrected *P* value <0.01). Non-redundant GO terms were obtained by retaining only one representative term from GO with high semantic similarity, derived using GOSemSim package^45^ in R.

### Detection of alternative splicing

Alternative splicing was detected at both transcript level and exon level. Differential splicing at the exon level was performed for 24 h samples (in hypoxia) versus 0 h using the DEXSeq package in R. DEXSeq uses transcript annotations of each gene to split them non-overlapping bins (exon counting bins) and then uses Generalized Linear Models to identify differentially used exon counting bins. Only exon counting bins that were at least 50-bp long, with mean exonic counts >1 and satisfied a FDR cutoff of 5% were considered as differentially used. Differential splicing at the transcript level was performed for 24-h samples (in hypoxia) versus 0 h using the Cuffdiff program within Cufflinks. In addition to detecting differential splicing, Cuffdiff also reports statistically significant differential promoter usage and differential coding sequence usage. Statistically significant changes were detected at a FDR cutoff of 5%. Following this, the alternative splicing events were annotated into 5 major categories, Exon Skipping, Mutually Exclusive Exons, Intron Retention, Alternative 3′ Splice Sites and Alternative 5′ Splice Sites, using MATS (3.0.8). The splicing events from MATS were detected using both read information and exon junction data and filtered for statistically significant events (FDR<1%).

### Analysis of TCGA data

Splice-aware aligned RNAseq data (BAM files) of Colorectal cancer cohort (COAD)^29^ comprising of 458 tumour samples and 41 normal samples along with corresponding clinical data was obtained from TCGA. Gene and transcripts quantification and normalisation was performed using the standard Cufflinks pipeline and the annotations in Ensembl (v74). In all cases exon-mapping reads substantially outnumbered intron-mapping reads: median exon/intron ratio per kb=557.5, with minimal difference between HiSeq 2000 and GAII processed samples (543.7 and 637.6, respectively).
