# Supplementary material for: Hypoxia-driven splicing into noncoding isoforms regulates the DNA damage response
Source: NPJ Genom Med. 2016 Jul 20;1:16020–. doi: 10.1038/npjgenmed.2016.20 (PMC5417364; doi:10.1038/npjgenmed.2016.20)
Supplement: Supplementary Information [file npjgenmed201620-s1.doc]

Sample QC

*In vitro data*

Samples were sequenced to an average depth of 43.8M (27.5M-58.2M) read-pairs, sufficient for splicing studies. >90% of sequenced reads were found to be uniquely mapping using a splice-aware aligner (Supplementary Table S1). Furthermore, more than 1/3^rd^ of reads fell at splice junctions, providing substantial evidence with which to identify splicing changes. We calculated the read distribution in different regions of the genome (exons, introns, UTRs and intergenic regions). The majority of reads fell within coding exons (mean = 2069 tags/kb), 5’ UTRs (174.1 tags/kb) and 3’ UTR (408.9 tags/kb), while only a very small proportion of reads mapped to introns (2.6 tags/kb), confirming the selectivity of the poly(A) prep and indicating the reliability of the raw sequence data.

In addition, the conservative filtering approach we took during the data analysis resulted in a novel junction call rate of less than 2%, further confirming the reliability of the underlying data.

*Isoform prediction*

We used Mapsplice to predict exon junctions and Cufflinks to assemble transcripts. Mapsplice has been recently found to perform particularly well when making splice site predictions and to be more conservative (and more specific) than other aligners, as shown in a recent independent review in Nature Methods (Engström et al. 2013, Nature Methods). Cufflinks, which has been shown to successfully identify alternative splicing changes across a number of studies (e.g. (Florea L, Song L and Salzberg SL. F1000Research 2013)).

Few splicing changes were called at 1 and 2 hours in hypoxia (relative to 0 hours). Since overall RNA quality and sample QC was high and consistent across the dataset, these samples provide a useful control and support the conclusion that the changes observed are biological in nature, and not simply artifacts from the analysis.

*Alignment jitters*

We focused on junction reads in the alignment files to identify the position at which the read split (1-101) across exons and whether there were any biases for read ends. Junctions were much more likely to be split at the mid-point (48-52 nt) than at the ends (1-8 nt, 93-100 nt). Furthermore, this distribution was similar for all types of junctions i.e. complete matching, partially matching (start or end but not both) or completely novel (Supplementary Figure 3). These data therefore confirm that the predictions are unlikely to be affected by alignment issues.
