# Supplementary material for: Hypoxia-driven splicing into noncoding isoforms regulates the DNA damage response
Source: NPJ Genom Med. 2016 Jul 20;1:16020–. doi: 10.1038/npjgenmed.2016.20 (PMC5417364; doi:10.1038/npjgenmed.2016.20)
Supplement: Supplementary Figure S1 [file npjgenmed201620-s2.pdf]

A

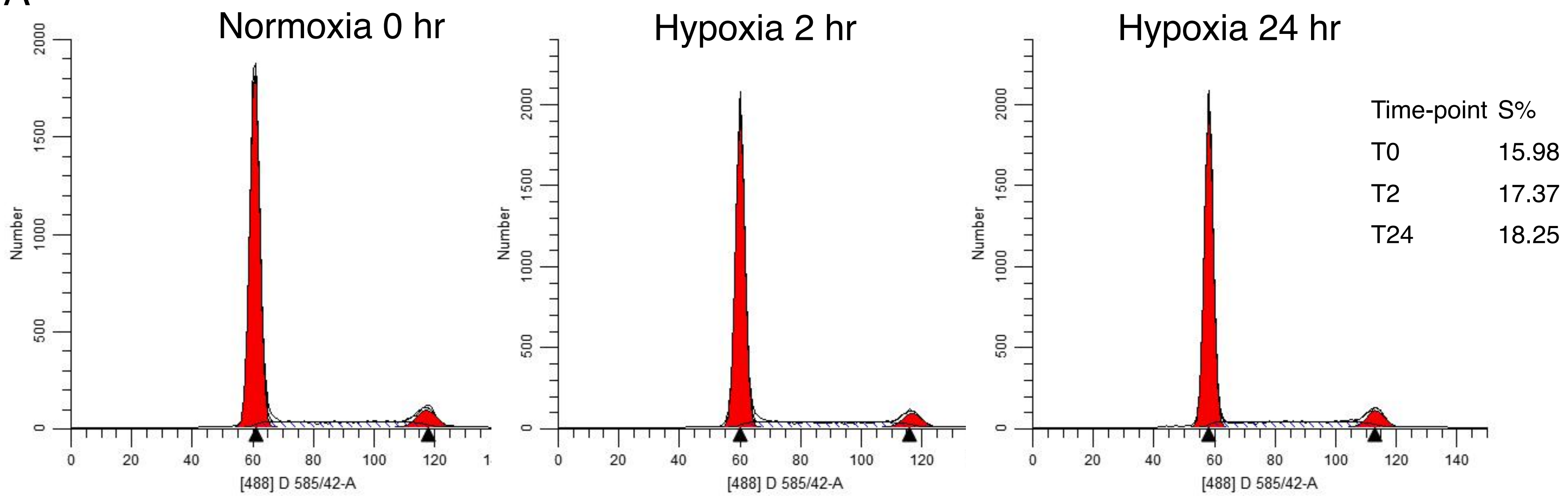

B

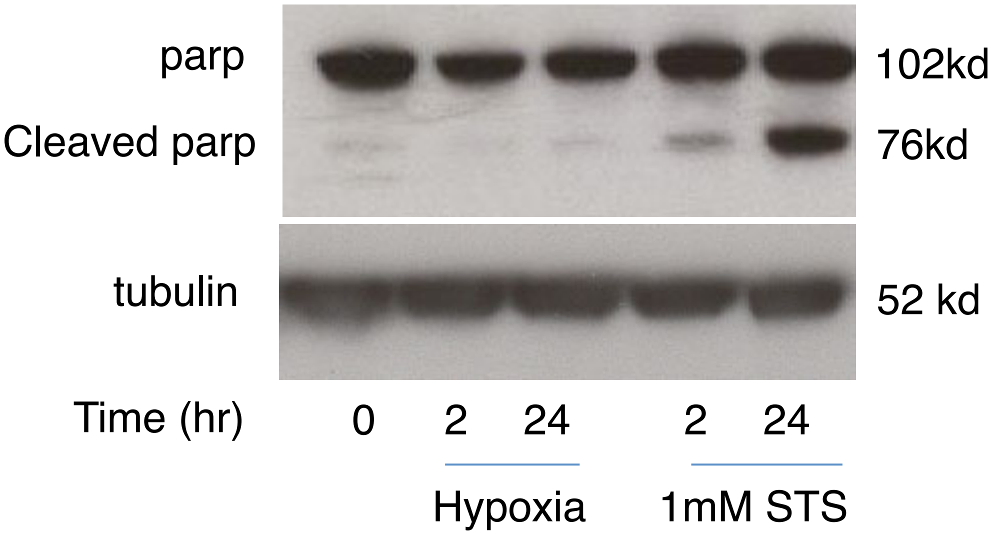

Figure S1 (A) No appreciable difference in cell cycle profile was observed in response to hypoxia. Methods as described in the main manuscript. **(B)** No appreciable activation of apoptosis was observed in response to hypoxia. Westerns were conducted as described in the main manuscript. Parp antibody: (Cell Signalling UK #9542). Positive apoptotic control: STS staurosporin (Fisher Scientific UK #10743783).
