# Supplementary material for: Hypoxia-driven splicing into noncoding isoforms regulates the DNA damage response
Source: NPJ Genom Med. 2016 Jul 20;1:16020–. doi: 10.1038/npjgenmed.2016.20 (PMC5417364; doi:10.1038/npjgenmed.2016.20)
Supplement: Supplementary Figure S2 [file npjgenmed201620-s3.pdf]

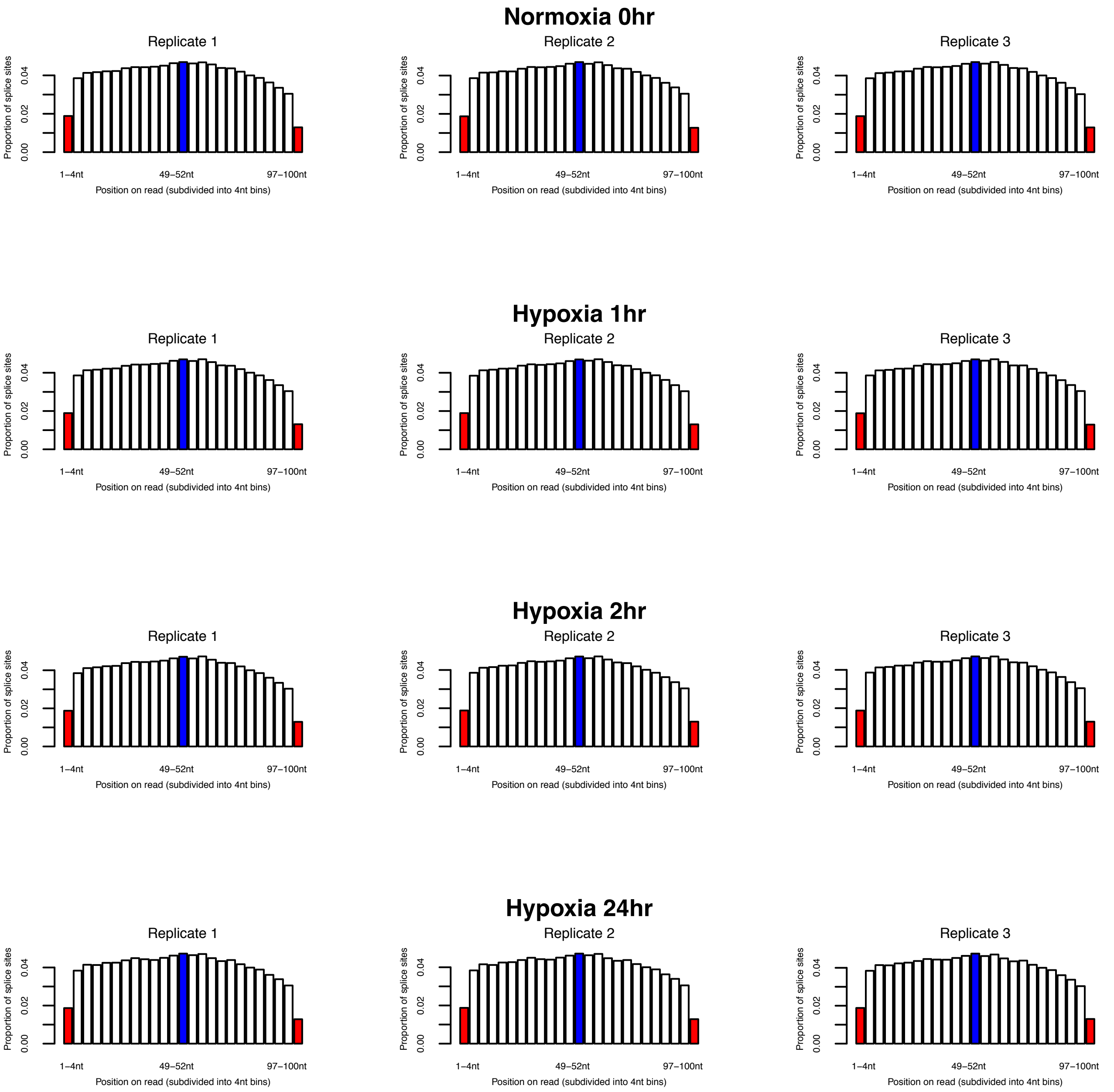

Figure S2

Distribution of position of splice sites on reads sub-divided into 4nt bins. Spliced reads were obtained from the BAM files and the position of splice junction on the read was identified.
