# Supplementary material for: Hypoxia-driven splicing into noncoding isoforms regulates the DNA damage response
Source: NPJ Genom Med. 2016 Jul 20;1:16020–. doi: 10.1038/npjgenmed.2016.20 (PMC5417364; doi:10.1038/npjgenmed.2016.20)
Supplement: Supplementary Figure S3 [file npjgenmed201620-s4.pdf]

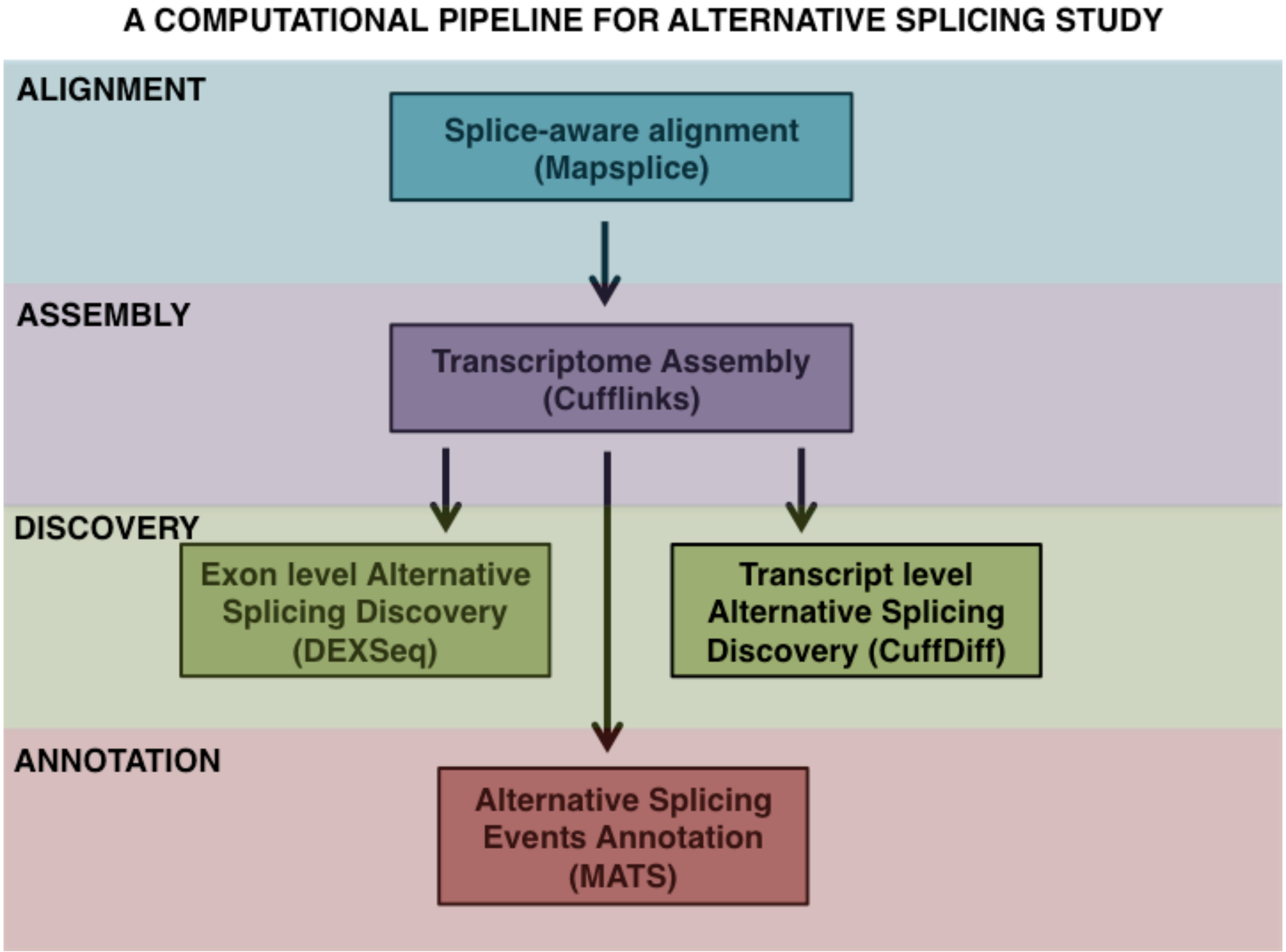

Figure S3
The pipeline used for discovery and annotation of alternative splicing events in hypoxia.
