# Supplementary material for: Hypoxia-driven splicing into noncoding isoforms regulates the DNA damage response
Source: NPJ Genom Med. 2016 Jul 20;1:16020–. doi: 10.1038/npjgenmed.2016.20 (PMC5417364; doi:10.1038/npjgenmed.2016.20)
Supplement: Supplementary Figure S4 [file npjgenmed201620-s5.pdf]

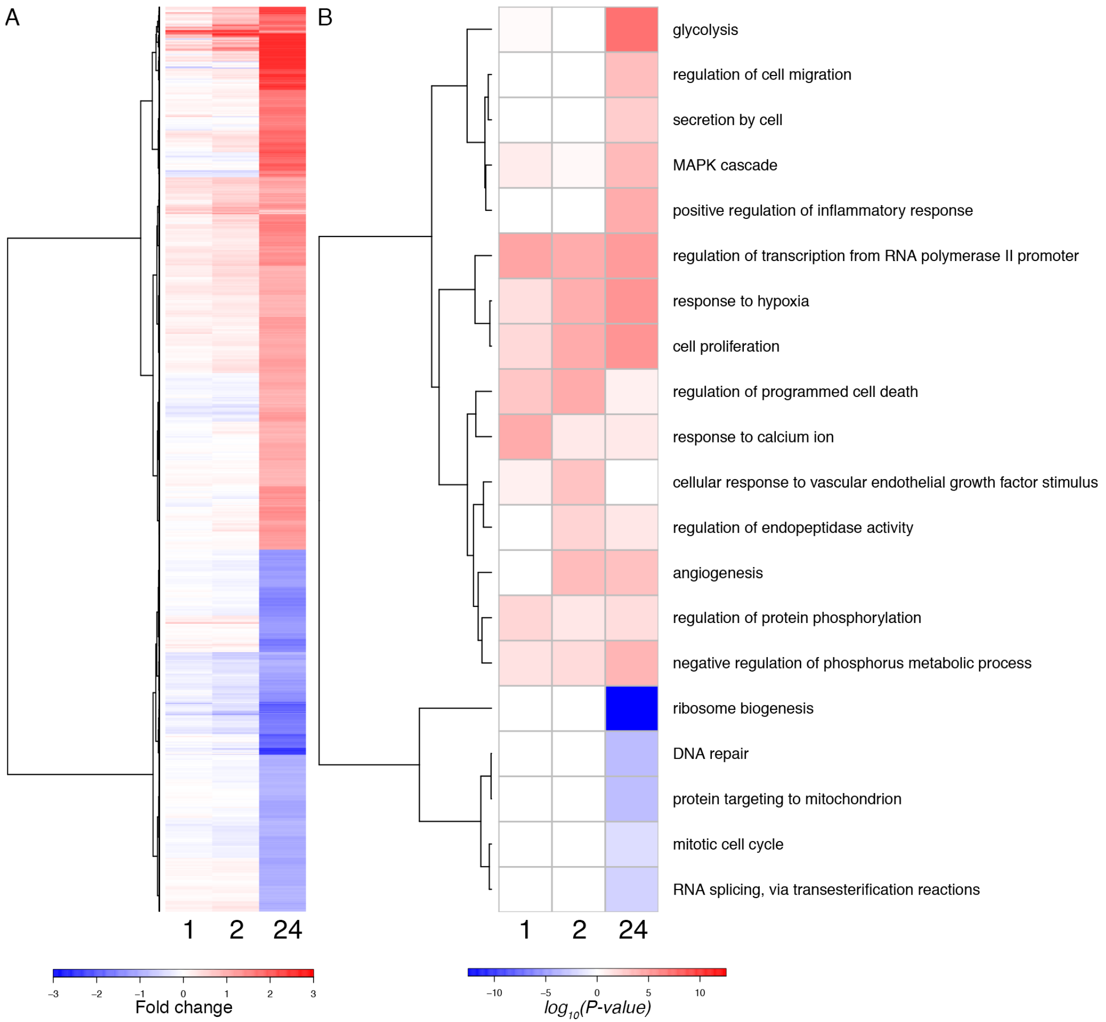

Figure S4

Global transcriptional changes for protein-coding loci in response to hypoxia. (A) Hypoxia-dependent differentially expressed protein-coding genes at 1, 2, or 24 hours. Rows represent the log2 fold changes (mean of three biological replicates) plotted relative to $t = 0$. Columns are ordered according to time point. (B) Key biological processes that are up- or down-regulated in response to hypoxia at 1, 2, or 24 hours. Rows represent the $-log_{10}(P\text{-}value)$ of up-regulated GO terms and $log_{10}(P\text{-}value)$ of down-regulated GO terms. Columns are ordered according to time point. Only non-redundant biological processes below a BH corrected *p*-value cutoff of 0.01 are represented. See also Table S2.
