# Supplementary material for: Hypoxia-driven splicing into noncoding isoforms regulates the DNA damage response
Source: NPJ Genom Med. 2016 Jul 20;1:16020–. doi: 10.1038/npjgenmed.2016.20 (PMC5417364; doi:10.1038/npjgenmed.2016.20)
Supplement: Supplementary Figure S5 [file npjgenmed201620-s6.pdf]

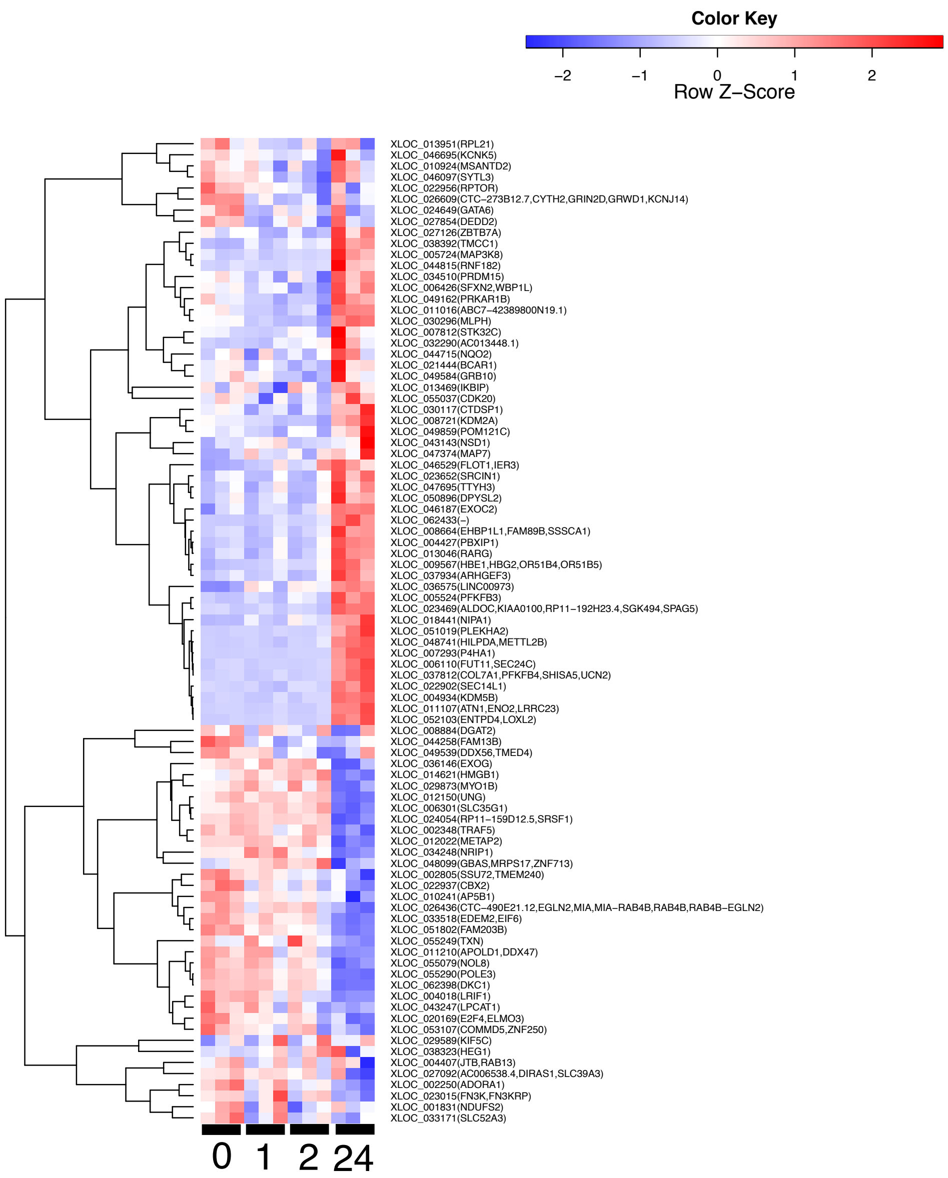


**Figure S5**

Expression profile of genes undergoing differential promoter usage in hypoxia. Rows represent genes, columns ordered by timepoint. Cells are coloured by the RPKM z-score.
