# Supplementary material for: Hypoxia-driven splicing into noncoding isoforms regulates the DNA damage response
Source: NPJ Genom Med. 2016 Jul 20;1:16020–. doi: 10.1038/npjgenmed.2016.20 (PMC5417364; doi:10.1038/npjgenmed.2016.20)
Supplement: Supplementary Figure S6 [file npjgenmed201620-s7.pdf]

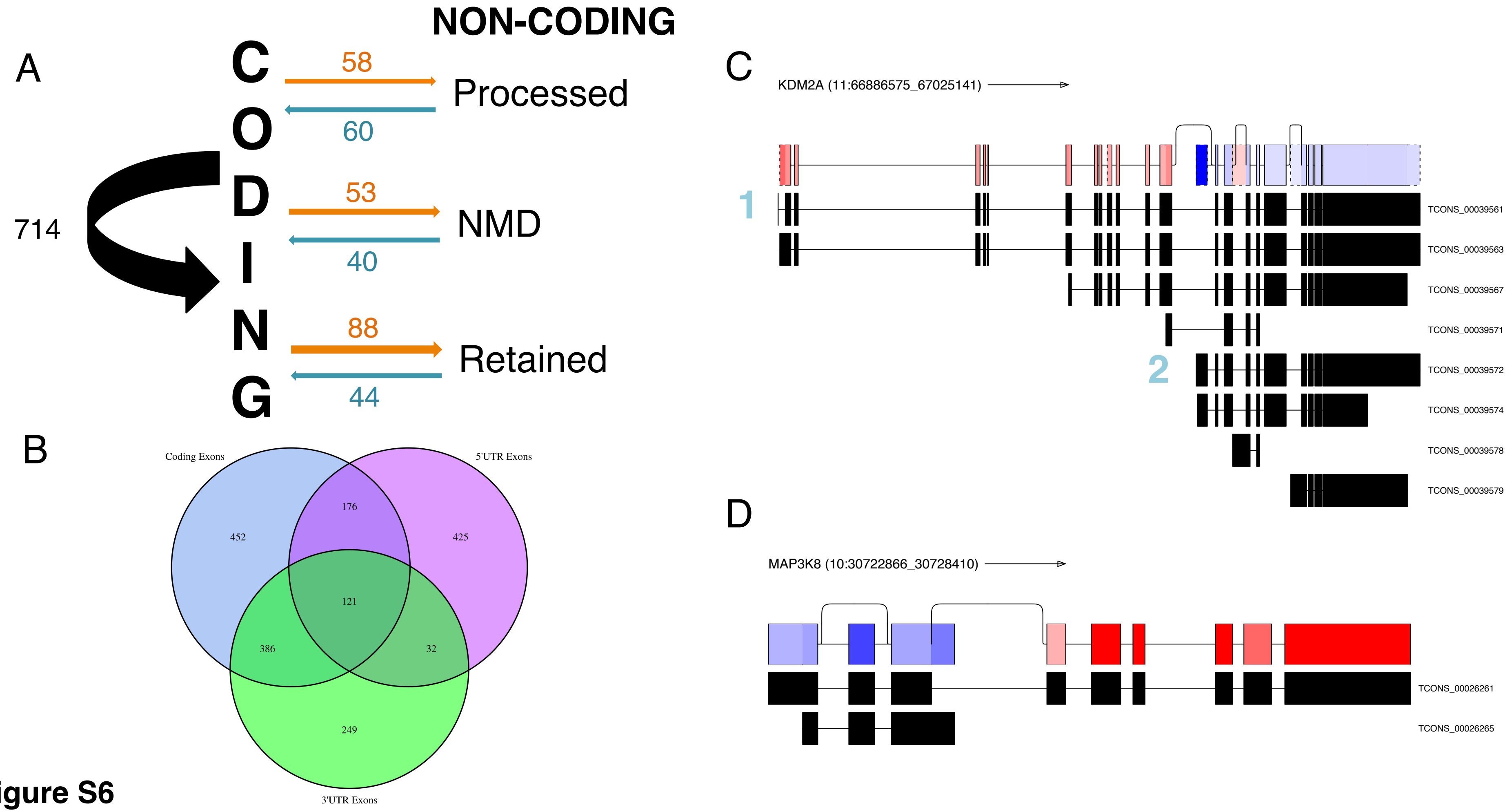

Figure S6

(A) Change in major isoform class between hypoxia and and normoxia samples; Orange: enriched in hypoxia samples. (B) No. of protein-coding genes which have differentially used exons at 24 hours in hypoxia relative to 0 hour time-point as identified by DEXSeq. The genes have been grouped according to exon annotations derived from Ensembl (v74) as either Coding Exons, 5' UTR and 3' UTR. (C) Predicted transcript structures are represented in black. Normalized fold changes between normoxia (blue) and hypoxia (red) are shown in color in the top row of each plot, with exon connectivity as determined from the RNA sequencing data used to generate exon links. (C) KDM2A. A novel alternate isoform of the lysine specific demethylase KDM2A lacking the Jumanji domain was expressed in normoxia (1). Elevated levels of the canonical full-length transcripts were observed in hypoxia (2). (D) A novel short isoform of MAP3K8/Tpl2/Cot Kinase missing the kinase domain was detected in normoxia, with the canonical full-length isoform being detected only in hypoxia.
